# Hospital characteristics related to the hospital length of stay among inpatients receiving invasive cervical discectomy due to road traffic accidents under automobile insurance in South Korea

**DOI:** 10.1186/s12913-017-2518-3

**Published:** 2017-08-16

**Authors:** Kyoung Won Shin, Hyo Jung Lee, Chung Mo Nam, Ki Tae Moon, Eun-Cheol Park

**Affiliations:** 1 Department of Health Policy and Management, Graduate School of Public Health, Yonsei University, Seoul, Republic of Korea; 2 Health Insurance Review and Assessment Service, Seoul, Republic of Korea; 3 Department of Public Health, Graduate School, Yonsei University, Seoul, Republic of Korea; 4 Institute of Health Services Research, Yonsei University College of Medicine, Seoul, Republic of Korea; 5 Department of Preventive Medicine, Institute of Health Services Research, Yonsei University College of Medicine, 50 Yonsei-ro, Seodaemun-gu, Seoul, 120-752 Republic of Korea; 6 National Evidence-Based Healthcare Collaborating Agency, Seoul, Republic of Korea

**Keywords:** Automobile insurance, Length of stay, Hospital characteristics, Moral hazard

## Abstract

**Background:**

In South Korea, people injured in road traffic accidents receive compensation for medical costs through their automobile insurance. However, the automobile insurance system appears to manage health care inefficiently. This study aimed to investigate the factors associated with the hospital length of stay (LOS), which was used as an indicator of healthcare utilization, for inpatients covered by automobile insurance and undergoing invasive cervical discectomy.

**Methods:**

Insurance claims data from 158 hospitals were used. The study included 850 inpatients who were involved in automobile accidents in 2014 and 2015 and who underwent invasive cervical discectomy. Poisson regression analysis was performed to examine the associations between the LOS and hospital-level characteristics.

**Results:**

The mean LOS for inpatients covered by automobile insurance was 25.75 days. A higher proportion of inpatients with automobile insurance were associated with a longer LOS (rate ratio [RR]: 1.027 per 1% increase, 95% confidence interval [CI]: 1.012–1.042). A higher hospital volume of invasive cervical discectomy (RR: 0.970 per 10 case increase, 95% CI: 0.945–0.997), bed turnover rate (RR: 0.988 per 1 increase, 95% CI: 0.979–0.997), and number of neurosurgeons or orthopedic specialists (RR: 0.930 per 1/100 beds increase, 95% CI: 0.876–0.987) were associated with a shorter LOS.

**Conclusions:**

Our findings suggest that inpatients covered by automobile insurance were associated with a longer LOS when treated at small-sized, low-provider, and low-volume hospitals with high proportions of such patients. Based on these findings, policymakers and healthcare professionals ought to consider improved strategies for efficient management of automobile insurance for inpatients in small-sized hospitals.

## Background

Transport accidents, most of which involve road traffic, are an important public health problem in South Korea, owing both to their high incidences and mortality rates. A traffic accident analysis showed that the mortality rate due to road traffic accidents was 10.1 people per 100,000 population in 2013; this rate was the third highest among Organization for Economic Cooperation and Development (OECD) countries, following Chile (12.0 per 100,000 population) and the United States (10.3 per 100,000 population). Additionally, the number of people injured due to road traffic accidents increased from 654.55 per 100,000 population in 2013 to 692.26 per 100,000 population in 2015 [1]. Since road traffic accidents mostly involve productive members of society, there are social costs involved as well [2]. Therefore, injured people should be treated promptly to accelerate their return to society.

In South Korea, people injured in road traffic accidents can receive compensation for medical costs through their automobile insurance, while people with illnesses receive National Health Insurance (NHI) coverage. Under the Guarantee of Automobile Accident Compensation Act, car owners are required to join an automobile insurance plan, and cars that are not insured are prohibited from operating [3]. However, even though automobile insurance is mandatory, the insurers are private companies that are governed by market drive and competition; as such, automobile insurance is a profit-seeking industry [4]. Although the medical costs generated by automobile insurance account for only 2.4% of the total medical costs [5], such insurance ought to be considered a part of the healthcare system because of its involvement in health-related coverage.

Furthermore, the automobile insurance system does not appear to manage healthcare efficiently. Previous studies have indicated a high rate of hospitalization and a long length of stay (LOS) for patients with automobile insurance [4, 6–8]. Cervical injuries are the most common cases handled by automobile insurance (48.4% of injured people); cervical sprains or contusions, which are regarded as minor injuries according to the Abbreviated Injury Scale, account for 98.5% of cervical injuries [9]. Additionally, a previous study showed that the percentage of patients with cervical sprains or contusions who were hospitalized under automobile insurance was 79.2%, which was much higher than that of 2.4% among patients covered by the NHI [10]. Moreover, the LOS of inpatients with the same injuries were longer for patients with automobile insurance than for those treated under the NHI [6, 11]. Studies in other countries have identified factors such as age, sex, insurance type, injury severity, hospital mortality, and hospital location as predictors of the LOS and hospital charges for road traffic injuries [12–14]. However, few studies have examined the factors affecting health care utilization, including the LOS and hospital charges, under automobile insurance [15, 16]. While health care utilization between the NHI and automobile insurance has been compared in previous studies [6, 7, 15], there are limited data on the LOS of patients under automobile insurance. In particular, there are no published analyses of hospital factors related to the LOS for patients with automobile insurance. Therefore, this study aimed to identify the hospital-related factors that are associated with the LOS, which was used as an indicator of health care utilization, in patients under automobile insurance.

## Methods

### Study population

We used two automobile insurance claims datasets acquired from the Health Insurance Review & Assessment Service (HIRA). Because the HIRA has been in charge of reviewing medical costs generated by patients with automobile insurance coverage from July 2013, we were able to access the first dataset including claims data for 850 patients who received invasive cervical discectomy between January 2014 and December 2015. We focused only on inpatients who underwent invasive cervical discectomy because of the variations in treatments for different injury types. Patients with automobile insurance were identified based on the electronic data interchange claim code that was provided during the review of reimbursement for healthcare services. The second dataset included aggregated claims data for each study year in terms of the medical institution utilization, such as the total number of hospitalizations and the total number of invasive cervical discectomies, by the NHI between January 2014 and December 2015. Additionally, reports on the status of the medical institutions were used in our study. These medical institution reports included general characteristics such as the number of beds, number of doctors, type of medical institution, and location. Of note, these data were based on a June 2016 report, which was beyond the study period, because these reports are updated periodically, and data from previous years were unavailable. All datasets were merged for the final analysis to assess the association between hospital characteristics and the LOS in inpatients who underwent invasive cervical discectomy under automobile insurance.

### Measures

To measure the outcomes of inpatients who underwent invasive cervical discectomy, we used the LOS as the outcome variable. The LOS is usually used as an indicator of efficiency or quality of hospital performance [17], and is calculated by subtracting the date of admission from the date of discharge.

The primary variables of interest in relation to the LOS were the hospital characteristics. Specifically, we included the proportion of inpatients with automobile insurance, volume of invasive cervical discectomy procedures, bed turnover rate, number of neurosurgeons or orthopedic specialists per 100 beds, number of beds, type of hospital, and location. The proportion of inpatients with car insurance was defined as the proportion of inpatients with automobile insurance among the total inpatients. The volume of invasive cervical discectomy refers to the total number of procedures performed within 1 year regardless of the health insurance type. The bed turnover rate was defined as the proportion of the total number of hospitalized patients by the number of beds. The numbers of neurosurgeons and orthopedic specialists were summed for each hospital, because both perform invasive cervical discectomies. The type of hospital was classified into hospital and general hospital. In South Korea, medical care organizations are divided into clinics and hospitals by function, and hospitals are further divided into hospitals and general hospitals by structural characteristics. Medical law defines clinics (less than 30 beds) as centers treating outpatients, and hospitals (more than 30 beds) as treating inpatients. General hospitals have more than 100 beds and at least 7 medical departments, including essential medical departments designated by medical law.

We also adjusted for inpatient characteristics when analyzing the relationship between hospital characteristics and the LOS. The included inpatient characteristics were sex, age, principal diagnosis, cervical spine surgery performed with cervical discectomy, Charlson Comorbidity Index (CCI), other surgical procedures performed during hospitalization, and the study period. The principal diagnosis was defined as the discharge diagnosis, as noted by the International Classification of Diseases codes, 10th revision. The CCI was calculated based on comorbid conditions at the time of inpatient admission to the hospital. Cervical spine surgeries performed with cervical discectomy were classified as none, arthrodesis, corpectomy, or corpectomy plus arthrodesis. Other surgical procedures during hospitalization included all procedures other than cervical surgery.

### Statistical analysis

We report the distribution of each categorical variable as the frequency and percentage; each continuous variable is reported as the mean and standard deviation. These analyses were performed for both inpatient-level and hospital-level variables. Next, analysis of variance (ANOVA) was performed for comparison of the mean (standard deviation) LOS according to each categorical variable. Third, we performed Poisson regression analysis after controlling for over-variance in order to examine both inpatient-level and hospital-level variables associated with the LOS. Subgroup analyses were also performed according to the study period, bed turnover rate, number of beds, and number of neurosurgeons/orthopedic specialists. The bed turnover rate, number of beds, and number of neurosurgeons/orthopedic specialists were categorized according to their median values. All statistical analyses were performed using SAS statistical software version 9.4. All calculated *P*-values were two-sided and considered significant at *P* < 0.05.

## Results

In this study, we analyzed 850 inpatients and 158 hospitals. Table 1 shows the general characteristics of the subjects; male inpatients were more frequent than female inpatients (78.1% vs. 21.9%, respectively). The mean age of the inpatients was 51.14 years. Most inpatients received arthrodesis at the same time as invasive cervical discectomy (91.9%). The mean proportion of inpatients with automobile insurance was 3.67%, and the mean volume of invasive cervical discectomy was 24.42. Additionally, the mean bed turnover rate was 32.07. There were more general hospitals (*n* = 118) than hospitals (*n* = 40) in our study. More hospitals were located in metropolitan areas (*n* = 107) than in non-metropolitan areas (*n* = 5).

**Table 1:** General characteristics of the study population

Variables	N/Mean	(%)/±SD
*Inpatient-level variables*		
Sex		
Male	664	(78.1)
Female	186	(21.9)
Age	51.14	±13.41
Principal diagnosis (ICD-10)		
Cervical disc disorders (M50)	103	(12.1)
Fracture of the neck (S12)	210	(24.7)
Dislocation, sprain and strain of joints and ligaments at the neck level (S13)	228	(26.8)
Injury of nerves and the spinal cord at the neck level (S14)	152	(17.9)
Others	157	(18.5)
Cervical spine surgery performed with cervical discectomy		
None	30	(3.5)
Arthrodesis	781	(91.9)
Corpectomy or corpectomy + arthrodesis	39	(4.6)
Charlson Comorbidity Index		
0	306	(36.0)
1	239	(28.1)
2	138	(16.2)
≥ 3	167	(19.7)
Other surgical procedures during hospitalization		
Not received	484	(56.9)
Received	366	(43.1)
Study period		
2014, January-June	251	(29.5)
2014, July-December	221	(26.0)
2015, January-June	210	(24.7)
2015, July-December	168	(19.8)
*Hospital-level variables*		
Proportion of inpatients with automobile insurance (%)	3.67	±3.36
Volume of invasive cervical discectomy	24.42	±25.26
Bed turnover rate	32.07	±7.26
Number of neurosurgeons/orthopedic specialists per 100 beds	2.56	±1.22
Number of beds	879.09	±476.82
Types of hospital		
Clinic or hospital (*N* = 40)	53	(6.2)
General hospital (*N* = 118)	797	(93.8)
Area		
Metropolitan (*N* = 107)	613	(72.1)
Non-metropolitan (*N* = 51)	237	(27.9)
Total	850	(100.0)

*ICD* International classification of diseases, *SD* standard deviation

Table 2 shows the results of the ANOVA for the LOS according to each categorical variable. The mean overall LOS per inpatient who underwent cervical discectomy was 25.75 days. Inpatients who underwent simple cervical discectomy had a shorter LOS compared with those who underwent more complicated procedures. Moreover, a higher CCI score was associated with a longer LOS. However, the LOS gradually decreased between the beginning of 2014 and the end of 2015. Patients in metropolitan area hospitals had a shorter LOS than those in non-metropolitan areas.

**Table 2:** Distribution of length of stay for each categorical variable

Variables	Length of stay		*p*-value^a^
	Mean	±SD	
*Inpatient-level variables*			
Sex			
Male	26.28	±22.39	0.2594
Female	23.83	±18.96	
Age			
Less than median (≤52)	23.50	±20.66	0.0011
More than median (>52)	28.37	±22.58	
Principal diagnosis (ICD-10)			
Cervical disc disorders (M50)	15.54	±12.28	<.0001
Fracture of neck (S12)	25.03	±20.21	
Dislocation, sprain and strain of joints and ligaments at neck level (S13)	22.66	±19.80	
Injury of nerves and spinal cord at neck level (S14)	32.51	±23.20	
Others	31.32	±25.78	
Cervical spine surgery performed with cervical discectomy			
None	17.27	±13.87	0.0049
Arthrodesis	25.65	±21.72	
Corpectomy or corpectomy + arthrodesis	34.26	±23.55	
Charlson Comorbidity Index			
0	21.22	±18.73	<.0001
1	24.87	±22.50	
2	28.36	±20.22	
≤ 3	33.14	±24.50	
Other surgical procedures during hospitalization			
Not received	20.95	±18.76	<.0001
Received	32.09	±23.62	
Study period			
2014, January-June	28.67	±23.62	0.0795
2014, July-December	25.15	±22.62	
2015, January-June	24.14	±19.30	
2015, July-December	24.16	±19.97	
*Hospital-level variables (cut-offs denote the median values)*			
Proportion of inpatients with automobile insurance (%)			
≤ 2.8%	23.09	±19.90	0.0016
> 2.8%	28.09	±23.14	
Volume of invasive cervical discectomy			
≤ 20.0	27.57	±21.43	0.0096
> 20.0	23.71	±21.83	
Bed turnover rate			
≤ 31.8	28.59	±23.59	<.0001
> 31.8	22.80	±19.12	
Number of neurosurgeons/orthopedic specialists per 100 beds			
≤ 2.2	28.24	±24.18	0.0025
> 2.2	23.63	±19.11	
Number of beds			
≤ 833	28.26	±21.15	0.0007
> 833	23.21	±21.97	
Types of hospital			
Clinic or hospital (*N* = 40)	20.06	±15.52	0.0646
General hospital (*N* = 118)	26.12	±22.00	
Area			
Metropolitan (*N* = 154)	24.74	±21.97	0.0007
Non-metropolitan (*N* = 71)	28.34	±20.80	
Total	25.75	±21.69	

*ICD* International classification of diseases, *SD* standard deviation

^a^The results of the analysis of variance

Table 3 shows the results of the Poisson regression analysis for the LOS considering both inpatient- and hospital-level variables. The CCI score showed a positive correlation with the LOS. However, the progress of time from early 2014 to late 2015 showed an inverse relationship with the LOS. In terms of hospital variables, hospitals with a higher proportion of inpatients covered by automobile insurance showed a positive correlation with the LOS. The hospital volume of invasive cervical discectomy showed an inverse relation with the LOS. Furthermore, the bed turnover rate was inversely correlated with the LOS. A greater number of neurosurgeons or orthopedic specialists was associated with a shorter LOS, as was a greater number of beds.

**Table 3:** Results of Poisson regression analysis^a^

Variables	Length of stay				
	RR^b^	95% CI			*P*-value
*Inpatient-level variables*					
Sex					
Male	1.000		-		
Female	0.974	0.871	-	1.090	0.6480
Age	0.997	0.993	-	1.002	0.2906
Principal diagnosis (ICD-10)					
Cervical disc disorders (M50)	1.000		-		
Fracture of neck (S12)	1.261	1.040	-	1.528	0.0182
Dislocation, sprain and strain of joints and ligaments at neck level (S13)	1.254	1.035	-	1.518	0.0207
Injury of nerves and spinal cord at neck level (S14)	1.509	1.242	-	1.835	<.0001
Others	1.642	1.352	-	1.995	<.0001
Cervical spine surgery performed with cervical discectomy					
None	1.000		-		
Arthrodesis	1.254	0.931	-	1.691	0.1367
Corpectomy or corpectomy + arthrodesis	1.674	1.180	-	2.374	0.0039
Charlson Comorbidity Index					
0	1.000		-		
1	1.227	1.072	-	1.404	0.0030
2	1.331	1.139	-	1.557	0.0003
≥ 3	1.543	1.285	-	1.853	<.0001
Other surgical procedures during hospitalization					
Not received	1.000		-		
Received	1.413	1.287	-	1.552	<.0001
Study period					
2014, January-June	1.000		-		
2014, July-December	0.912	0.811	-	1.027	0.1278
2015, January-June	0.790	0.697	-	0.896	0.0002
2015, July-December	0.768	0.671	-	0.879	0.0001
*Hospital-level variables*					
Proportion of inpatients with automobile insurance (%)	1.027	1.012	-	1.042	0.0005
Volume of invasive cervical discectomy (per 10 case increase)	0.970	0.945	-	0.997	0.0224
Bed turnover rate	0.988	0.979	-	0.997	0.0076
Number of neurosurgeons or orthopedic specialists per 100 beds	0.930	0.876	-	0.987	0.0161
Number of beds	0.977	0.964	-	0.990	0.0007
Types of hospital					
Hospital (*N* = 40)	1.000		-		
General hospital (*N* = 118)	1.205	0.930	-	1.562	0.1585
Area					
Metropolitan (*N* = 107)	1.000		-		
Non-metropolitan (*N* = 51)	0.997	0.895	-	1.110	0.9520

*ICD* International classification of diseases, *RR* rate ratio, *CI* confidence interval

^a^All variables in the table were simultaneously adjusted

^b^The RRs (rate ratios) indicate the results of exponentiated coefficients and can be interpreted as percentage changes

**Fig. 1:**
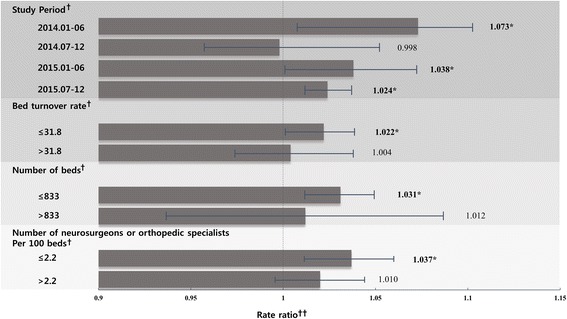
Association between the proportion of inpatients with automobile insurance and the length of stay according to the study period, bed turnover rate, number of beds, and number of neurosurgeons/orthopedic specialists﻿. † The results were calculated by Poisson regression analysis adjusted for inpatient-level and hospital-level characteristics. †† The rate ratios (RRs) indicate the results of the exponentiated coefficients and can be interpreted as the percentage changes. * Indicates that the results were statistically significant (*P* < 0.05)

We also performed subgroup analyses according to the study period and the median hospital bed turnover rate, number of beds, and number of neurosurgeons/orthopedic specialists to investigate the relationship between the proportion of inpatients with automobile insurance and the LOS. The proportion of inpatients with automobile insurance showed a positive relationship with the LOS in each study period except for that between July and December 2014. Furthermore, the proportion of inpatients with automobile insurance showed a positive correlation with the LOS only at institutions where the bed turnover rate, number of beds, and number of neurosurgeons/orthopedic specialists were below their respective median values (Fig. 1).

## Discussion

South Korean hospital patients have a longer mean LOS than patients in other countries. According to OECD health data, the mean LOS per inpatient in South Korea was twice as long as that in OECD countries overall in 2013 (South Korea: 16.5 days; all OECD countries: 8.1 days); accordingly, South Korea had the second longest LOS duration (Japan had the longest LOS duration) [18]. A longer mean LOS increases the medical costs of patients during hospitalization, and delays the transition from inpatient to outpatient treatment [19]. Thus, the longer mean LOS in South Korea can be interpreted to mean that medical resources are not being used efficiently. In particular, a longer LOS of inpatients with automobile insurance (as compared to those with NHI coverage) has been found in a few previous studies [6, 7, 15]; however, these studies are insufficient considering the extent of the problem. Therefore, the current study aimed to further assess the relationship between hospital characteristics and the LOS in inpatients who are covered under automobile insurance in order to provide justification for better management of patients with hospital stay and to improve policy measures under the automobile insurance system.

Because cervical injuries represent one of the most common conditions related to automobile accidents, and as there are several different treatments of cervical injuries, we focused on invasive cervical discectomies in the present study. Our findings suggest that higher volumes of invasive cervical discectomy, bed turnover rate, number of neurosurgeons/orthopedic specialists, and number of beds were associated with a shorter LOS for patients with automobile insurance coverage. These results were consistent with those of previous studies [20–24]. Hospitals that treat more cases may have highly skilled surgeons who specialize in the required procedures, as well as more consistent processes for postoperative care, better-staffed intensive care units, and greater general resources for dealing with postoperative complications [20, 21]. Furthermore, it has been reported that a higher number of surgeons was independently associated with a significantly decreased rate of postoperative complications, a significantly shorter LOS, and significantly lower costs [22].

Interestingly, hospitals with a higher proportion of inpatients with automobile insurance were associated with a longer LOS in our study; this may be explained by the features of the automobile insurance process in Korea, where the health care payment systems of health insurance and automobile insurance are unified; this differs from that in other countries. First, inpatients with automobile insurance have no copayment, because the perpetrator of the traffic accident is responsible for paying. Thus, patients are more likely to undergo unnecessary treatment or be over-diagnosed. Second, medical costs vary between health insurance providers, even in patients with the same symptoms. There is a medical cost difference between tertiary hospitals (where automobile insurance-based charges are 15% higher than NHI-based charges) and general hospitals (where automobile insurance-based charges are 12% higher than NHI-based charges). However, there is little or no difference between hospitals (where automobile insurance-based charges are 1% higher than NHI-based charges) and clinics (where charges are equivalent). Additionally, there is a difference in the cost of inpatient care per day according to the LOS. For those with NHI coverage, a longer LOS decreases the daily cost of inpatient care, regardless of the type of medical institution. However, there is no such decrease in cost with a longer LOS for patients under automobile insurance in tertiary and general hospitals. In hospitals and clinics, the cost of inpatient care per day for those with automobile insurance decreases after 50 days of hospitalization, while for those under the NHI, the daily cost decreases after 16 days. Previous studies suggested that health insurance might increase utilization due to supply-induced demand, especially under a fee-for-service system in which providers have a financial incentive to perform additional medical and surgical procedures [25, 26]. Furthermore, consumer-induced demand may also exist, because consumers use healthcare services without copayments. Many previous studies have reported that greater insurance coverage benefits imply less risk-bearing by the insured but result in a greater moral hazard [27–31].

The type of hospital was not significantly associated with the LOS in our study. Furthermore, the association between a higher proportion of inpatients with automobile insurance and a longer LOS was significant only in hospitals with lower bed turnover rates, numbers of beds, and numbers of neurosurgeons/orthopedic specialists. The management of the LOS in each hospital can be categorized into two strategies based on the hospital characteristics and performance. One on hand, hospitals strive to increase the LOS because the medical profits increase per inpatient day. Under fee-for-service systems, small-sized, low-performance hospitals are often motivated to create profits through a longer LOS. This motivation appears to be particularly evident in hospitals with higher proportions of inpatients with automobile insurance. On the other hand, most large-sized hospitals tend to strive to reduce the LOS, because they already have high hospital occupancy and prefer to increase the revenue by accelerating patient turnover rates.

Previously, reviewing medical costs generated by automobile insurance-covered inpatients was the responsibility of the respective insurance companies. The South Korean government instructed the HIRA to review medical costs generated by patients with automobile insurance coverage from July 2013 onwards in order to manage the LOS of the patients and analyze the increase in medical costs. Owing to this government intervention, the LOS of inpatients with automobile insurance has decreased over time (Table 3). However, the moral hazard still appears to exist, as shown in our subgroup analyses of different time periods. Given our results, health policymakers ought to consider proper strategies for effective management of small-sized hospitals. The quality of medical evaluations performed by the NHI should also apply to automobile insurance, and an objective evaluation system, based only on medical evidence, should be developed to ensure appropriate treatment for all patients.

This study has some advantages compared to previous studies on the topic. First, to our knowledge, this is the first study using claims data from the HIRA, which include all inpatients undergoing invasive cervical discectomy under automobile insurance in South Korea. Second, the data used in our study take into account the hierarchical nature of claims data, and can therefore capture the diversity of both inpatients and hospitals. Such data are especially helpful in establishing evidence-based health policies. Therefore, our results may prove useful for designing an effective strategy for managing patients with automobile insurance.

However, our study also has some limitations related to limited data or methodological issues. First, the current study included only inpatients undergoing invasive cervical discectomy. Therefore, our results may not be applicable to those with other medical conditions that are covered by automobile insurance. Additionally, hospital information (i.e., the number of neurosurgeons or orthopedic specialists and the number of beds) was based on June 2016 data because of limited access to older data; meanwhile, the patient data were extracted in 2014-2015. However, other hospital parameters, including the proportion of inpatients with automobile insurance, volume of invasive cervical discectomy, and bed turnover rate, were derived from the same period as the patient data. Third, we could not identify cases of multiple hospitalizations for a single inpatient, because the data used in our study comprised only information on cases of hospitalization for invasive cervical discectomy. Fourth, there may be several other unexplored factors that can affect the LOS; however, we were unable to consider more detailed variables owing to data limitations. Finally, our findings may not be applicable to other countries because of the specific automobile insurance policies in South Korea.

Despite these limitations, the identification of associations between certain hospital characteristics and the LOS under automobile insurance has implications for both future studies and for healthcare system planning related to automobile insurance. Given that we focused on the LOS, future studies should explore whether hospital characteristics and hospital charges are linked in order to identify the healthcare utilization of automobile insurance-covered inpatients. In addition, the associations between hospital characteristics and the LOS of inpatients with other medical conditions under automobile insurance need to be evaluated to determine whether our results are generalizable. Our findings may also aid in future policymaking to design efficient management systems of automobile insurance. Furthermore, our study could serve as a basis for healthcare professionals in other countries for reforming healthcare insurance systems, including automobile insurance systems.

## Conclusions

Our findings suggest that a higher proportion of inpatients with automobile insurance is associated with a longer LOS in small-sized, low provider- and procedure-volume hospitals. Based on these findings, health policymakers and healthcare professionals should consider proper strategies for the effective management of patients under automobile insurance treated in small-sized hospitals.

## Abbreviations

ANOVA: Analysis of variance
CCI: Charlson comorbidity index
HIRA: Health Insurance Review & Assessment Service
LOS: Length of stay
NHI: National Health Insurance
OECD: Organization for Economic Cooperation and Development
RR: Rate ratio

## References

[1] Traffic Accident Analysis System: Mortality of road traffic accident in OECD countries; 2013.

[2] Balu S, Simko RJ, Quimbo RM, Cziraky MJ (2009). Impact of fixed-dose and multi-pill combination dyslipidemia therapies on medication adherence and the economic burden of sub-optimal adherence. Curr Med Res Opin.

[3] The Ministry of Land, Infrastructure and Transport: Guarantee of automobile accident compensation act; 1963.

[4] Research Institute for Healthcare Policy (2006). Suggestion on the improvement of car insurance system: focused on medical field.

[5] Health Insurance Review & Assessment Service (2016). Statistics of medical expense in 2015.

[6] Kim JD, Jeong EW, Lee JH (2012). Study on the unification theory of added-ratio by of medical institution between national insurance and traffic accident insurance. Health Ser Manag Rev.

[7] Yoo H-S, Park T-S (2007). An analysis of automobile insurance and health insurance patients’ uses of the medical services. Korean J Prev Med Soc.

[8] Lee Y-J (2011). Problems of national medical expenses management in Korea. J Korea Contents Assoc.

[9] Jeong J-I (2013). A study on medical charge assessment contract for auto insurance: focused on clause 2, article 12, motor vehicle compensation guarantee act. J Law Res.

[10] Ministry of Land, Transport and Maritime Affairs: Basic research for improving health care system of auto insurance; 2011.

[11] Kwon CI, Kim JH, Kim JH, Lee S-H (2007). Social-economic effects of integrating insurance review system: National Health Insurance, automobile insurance and work accident insurance. Health Soc Sci.

[12] Haghparast-Bidgoli H, Saadat S, Bogg L, Yarmohammadian MH, Hasselberg M (2013). Factors affecting hospital length of stay and hospital charges associated with road traffic-related injuries in Iran. BMC Health Serv Res.

[13] Peek-Asa C, Yang J, Ramirez M, Hamann C, Cheng G (2011). Factors affecting hospital charges and length of stay from teenage motor vehicle crash-related hospitalizations among United States teenagers, 2002–2007. Accid Anal Prev.

[14] Gardner R, Smith GA, Chany A-ML, Fernandez SA, McKenzie LB (2007). Factors associated with hospital length of stay and hospital charges of motor vehicle crash–related hospitalizations among children in the United States. Arch Pediatr Adolesc Med.

[15] Jin J-H, Oh M-A (2013). Data analysis of hospitalization of patients with automobile insurance and health insurance: a report on the patient survey. J Korean Data Anal Soc.

[16] Ko M-S, Choi J-Y, Kim S-H (2011). Factors affecting medical treatment and expenses for the inpatients under coverage of car insurance by traffic accident. J Korea Contents Assoc.

[17] Thomas JW, Guire KE, Horvat GG (1997). Is patient length of stay related to quality of care?. J Healthc Manag.

[18] OECD (2015). Health at a glance 2015: OECD indicators.

[19] Hong S-O, Park J-H, Kim Y-T, Kang S-H (2015). The variation of factors of severity-adjusted length of stay in injury of neck. Korea Inst Health Soc Aff.

[20] Birkmeyer JD, Siewers AE, Finlayson EV, Stukel TA, Lucas FL, Batista I, Welch HG, Wennberg DE (2002). Hospital volume and surgical mortality in the United States. N Engl J Med.

[21] Han K-T, Lee HJ, Park E-C, Kim W, Jang S-I, Kim TH (2016). Length of stay and readmission in lumbar intervertebral disc disorder inpatients by hospital characteristics and volumes. Health Policy.

[22] Dasenbrock HH, Clarke MJ, Witham TF, Sciubba DM, Gokaslan ZL, Bydon A (2012). The impact of provider volume on the outcomes after surgery for lumbar spinal stenosis. Neurosurgery.

[23] Joynt KE, Orav EJ, Jha AK (2011). The association between hospital volume and processes, outcomes, and costs of care for congestive heart failure. Ann Intern Med.

[24] Nguyen Y-L, Wallace DJ, Yordanov Y, Trinquart L, Blomkvist J, Angus DC, Kahn JM, Ravaud P, Guidet B (2015). The volume-outcome relationship in critical care: a systematic review and meta-analysis. CHEST J.

[25] Sepehri A, Simpson W, Sarma S (2006). The influence of health insurance on hospital admission and length of stay—the case of Vietnam. Soc Sci Med.

[26] Rohrer JE (1990). Supply-induced demand for hospital care. Health Serv Manag Res.

[27] Trottmann M, Zweifel P, Beck K (2012). Supply-side and demand-side cost sharing in deregulated social health insurance: which is more effective?. J Health Econ.

[28] Newhouse JP (1996). Reimbursing health plans and health providers: efficiency in production versus selection. J Econ Lit.

[29] Zweifel P, Manning WG (2000). Moral hazard and consumer incentives in health care. Handb Health Econ.

[30] Ellis RP, McGuire TG (1993). Supply-side and demand-side cost sharing in health care. J Econ Perspect.

[31] Albert Ma CT, Riordan MH (2002). Health insurance, moral hazard, and managed care. J Econ Manag Strateg.

